# Challenges and insights in immunization in patients with demyelinating diseases: a bench-to-bedside and evidence-based review

**DOI:** 10.1590/0004-282X-ANP-2022-S121

**Published:** 2022-08-12

**Authors:** Guilherme Diogo Silva, Vítor Falcão de Oliveira, Leonardo Oliveira Mendonça

**Affiliations:** 1 Universidade de São Paulo, Faculdade de Medicina, Hospital das Clínicas, Departamento de Neurologia, São Paulo SP, Brazil.; 2 Universidade de São Paulo, Faculdade de Medicina, Hospital das Clínicas, Departamento de Moléstias Infecciosas e Parasitárias, São Paulo SP, Brazil.; 3 Universidade de São Paulo, Faculdade de Medicina, Hospital das Clínicas, Departamento de Imunologia Clínica e Alergia, São Paulo SP, Brazil.; 4 Rede DASA-Hospital 9 de Julho, Divisão de Imunologia Clínica e Alergia, São Paulo SP, Brazil.; 5 Rede DASA-Hospital 9 de Julho, Centro de Doenças Raras e da Imunidade, São Paulo SP, Brazil.

**Keywords:** Vaccines, Demyelinating Diseases, Multiple Sclerosis, Immunization, Neuromyelitis Optica, Vacinas, Doenças Desmielinizantes, Esclerose Múltipla, Imunização, Neuromielite Óptica

## Abstract

**Background:**

Infections are among the main causes of death in patients with demyelinating diseases of the central nervous system (CNSDD). Vaccines are effective methods in reducing hospitalization and death from infectious diseases, but they are challenging in patients with CNSDD because of autoimmunity and immunosuppression.

**Objectives:**

To summarize the pathophysiological rationale and main evidence for vaccine recommendations in patients with CNSDD.

**Methods:**

Specialists with different backgrounds on the subject: a neurologist specialized in demyelinating diseases, an infectious diseases specialist and an immunologist, presented a critical narrative review of vaccination literature in patients with CNSDD, highlighting which vaccines should or should not be administered and the best time for it.

**Results:**

Patients with DDSNC are at increased risk of vaccine-preventable viral and bacterial infections. Vaccines can prevent herpes zoster, hepatitis B reactivation, HPV-associated warts and tumors, viral and bacterial pneumonia, and meningitis. Live attenuated virus vaccines should not be used when the patient is on immunosuppression. Vaccines should be avoided during relapses. The greatest vaccine efficacy is given before treatment or at the end of medication.

**Conclusion:**

Patients with DDSNC need differentiated immunization in relation to additional vaccines, contraindicated vaccines and timing of vaccination.

## RESUMO

**Antecedentes:**

Infecções estão entre as principais causas de morte de pacientes com doenças desmielinizantes do sistema nervoso central (DDSNC). Vacinas são métodos eficazes para reduzir internação e morte por doenças infecciosas, porém são desafiadoras em pacientes com DDSNC tanto pela autoimunidade quanto pela imunossupressão.

**Objetivos:**

Resumir o racional fisiopatológico e as principais evidências para as recomendações de vacinas em pacientes com DDSNC.

**Métodos:**

Especialistas com diferentes formações no tema: um neurologista especialista em doenças desmielinizantes, um infectologista e um imunologista, apresentam uma revisão crítica narrativa da Literatura de vacinação em pacientes com DDSNC, com destaque a quais vacinas devem ou não ser administradas e o melhor momento para isso.

**Resultados:**

Pacientes com DDSNC têm risco aumentado para infecções imunopreveníveis virais e bacterianas. Vacinas podem prevenir herpes zooster, reativação de hepatite B, verrugas e tumores associados ao HPV, pneumonias virais e bacterianas, além de meningites. Vacinas de vírus vivos atenuados não devem ser usadas quando o paciente está em uso de imunossupressão. Vacinas devem ser evitadas durante surtos. A maior eficácia vacinal é dada antes do tratamento ou ao final de doses de medicações.

**Conclusão:**

Os pacientes com DDSNC necessitam de imunização diferenciada em relação a vacinas adicionais, vacinas contraindicadas e melhor momento de vacinação.

## INTRODUCTION

 Central Nervous System Demyelinating Diseases (CNSDD) are a heterogenous group of disorders with an acute or chronic inflammatory immunological process. This group of disorders is composed of the two most common forms, multiple sclerosis (MS) and neuromyelitis optica spectrum disorders (here referred to as NMO) and also more rare conditions such as acute disseminated encephalomyelitis and myelin oligodendrocyte glycoprotein antibody-associated disease. Patients with CNSDD have been found to be at an increased risk of infectious diseases in large observational retrospective studies^1^. Moreover, infections are the leading cause of death in patients with multiple sclerosis^2^ and neuromyelitis optica^3^.

Vaccines are an effective strategy to promote striking decreases in hospitalizations and deaths associated with infectious-preventable diseases^4^. However, immunization in patients with CNSDD is complex. The disease *per se* and the immunosuppressive treatment can cause physicians and patients concerns such as precipitation of disease flares, severe adverse events after live attenuated vaccines, reduction of vaccine effectiveness, and the need for additional vaccines in a population with increased risk of infections^5^.

In this review, we aim to propose a simplified approach, based on the actual evidence-based recommendations, experimental evidence, and the experience from experts from different specialties to guide the physicians who care for patients with CNSDD.

## METHODS

Experts with different backgrounds in the field of vaccination in CNSDD patients provided a summary of relevant literature: a neurologist specialized in demyelinating diseases, an infectious diseases specialist and an immunologist. Articles were selected based on the experience of the experts and a non-systematic literature review using the terms: "vaccination", "immunization", "multiple sclerosis", "neuromyelitis optica", "demyelinating diseases" in Pubmed, Embase and Scopus. 

For didactic reasons, we subdivided this review into sub-topics: additional vaccines that should be administered, vaccines that should not be administered, and the best time to administer the immunization. 

## RESULTS

### Which vaccines should be administered in patients with CNSDD?

Patients with multiple sclerosis and neuromyelitis optica present with dysregulated immune-mediated responses that damage the CNS. Disease-modifying drugs (DMDs) can reduce the autoimmune response at the cost of harnessing the normal response against viral and bacterial agents. The two prototypical CNSDD are multiple sclerosis and neuromyelitis optica, this being the reason why we have focused our review-based analysis on these^6^.

The autoimmune theory of the immunological response in patients with multiple sclerosis is widely studied and is characterized by the myelin self-reactive T-CD4 cells that migrate from the periphery to the CNS (also known as. the outside-in theory)^7^. These cells activate macrophages and microglia, leading to the damage of neurons and oligodendrocytes. This process leads to an epitope spreading, a process in which new myelin antigens are exposed, enhancing the theoretical autoimmune response (also known as the inside-out theory)^7^. Based on this theory, the treatment of multiple sclerosis involves drugs that target the aberrant immunological process of the self-reactive T-cells. Said that, drugs used are those that alter cytokine response (e.g. interferon beta), block myelin-specific antigen presentation (e.g. glatiramer acetate), block leukocyte migration into the CNS (e.g. S1P receptor modulators - fingolimod, siponimod, and posanimod - and cell adhesion agents - natalizumab), block B-cell dependent T-cell activation (e.g. anti-CD20 therapies - rituximab, ocrelizumab, and ofatumumab) or lead to B and T cell depletion (e.g. anti-CD52 strategies - alemtuzumab - or non-selective cytotoxic treatments - cladribine)^8^.

The autoimmune theory in patients with neuromyelitis optica is supported by the presence of serum-detectable levels of anti-aquaporin-4 autoantibodies that target astrocytes, culminating with the activation of the complement system and enhanced cytokine production (e.g. interleukin-6), considered the main mechanism of CNS damage^9^. As previously said, the treatment of neuromyelitis optica includes anti-CD20 (e.g. rituximab) and anti-CD19 (e.g. inebilizumab) drugs that suppress the anti-aquaporin-4 autoantibodies production by B-cells; anti-C5 drugs (eculizumab) that inhibits the complement system; and anti-IL-6 (satralizumab or tocilizumab) that mitigates this cytokine production. Non-specific cytotoxic agents such as azathioprine, methotrexate and mycophenolate, considered to non-specifically block T-self reactive cells, are also used in these diseases^10^.

We highlight the importance of five viral vaccine-preventable agents in patients with CNS demyelinating diseases: varicella, hepatitis B, human papillomavirus (HPV), influenza and SARS-CoV-2.

**Figure 1.**
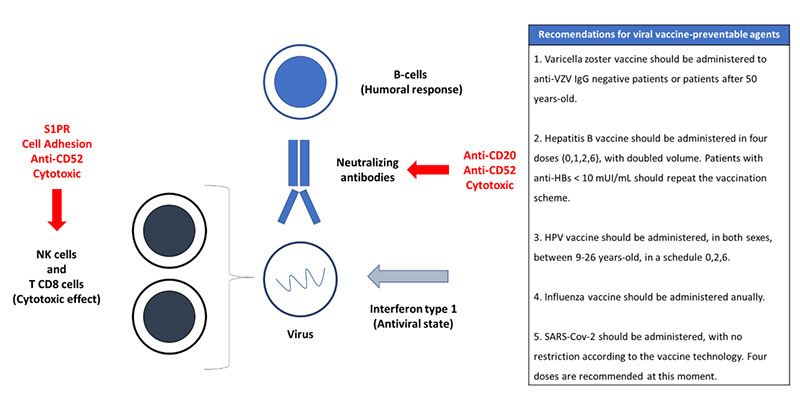
Normal immune response to viral agents, how disease-modifying drugs impact this response and what vaccine strategies we should implement.

The normal immune response to viral agents initiates with the activation of the innate immunity by the production of the interferon-type 1, the induced antiviral state followed by the viral neutralization mediated by antibodies and the cytotoxic killing of the infected cells by natural killer cells and TCD8 lymphocytes^11^. Any inherited or induced perturbations in the immunological process referred to here that allows viral escape of the immune response may cause life-threatening infections. Figure 1 summarizes the normal immune response to viral agents, the impact of DMDs and vaccine recommendations for clinical practice^12^.

As interferon-beta is a type-1 interferon, it is not expected that this DMD used in the treatment of multiple sclerosis compromises the normal antiviral response. Glatiramer acetate blocks the antigen presentation of myelin and it is not expected to compromise the normal immune response to the virus^12^.

On the other hand, patients with multiple sclerosis are at an increased risk of the use of herpes antiviral use in population studies^1^, particularly for patients using sphingosine-1 phosphate (S1P) receptor modulators such as fingolimod.

S1P receptor modulators have an anti-trafficking cell effect that results in an accumulation of lymphocytes in secondary immune lymph tissues and induces peripheral blood lymphopenia^13^. Consequently it causes reduction of the VZV-specific T-cells inducing a subclinical reactivation in around 20% of the patients^14^ and symptomatic infection was found to occur in 11 per 1000 patients^15^.

Current guidelines recommend that a dose of the varicella zoster vaccine should be administered in individuals with varicella IgG negative antibodies and in those without a history of varicella infection^15^. However, varicella zoster vaccine is a live attenuated virus vaccine and should be administered four to six weeks before the initiation of immunotherapy in order to avoid severe adverse events related to the vaccine.

Although to a lesser extent when compared with S1P receptor modulators, patients under rituximab and natalizumab therapy also present an increased risk of herpes antiviral use than the general population^1^. These monoclonal antibodies (anti-CD20, rituximab) seem to predispose to the risk of varicella-zoster virus infection as they reduce the humoral response against VZV^16^ ^,^ ^17^. Cell adhesion inhibitors (natalizumab) compromise the cellular (CD4 and CD8 lymphocytes) surveillance in the CNS and may predispose not only to progressive multifocal leukoencephalopathy, a John Cunningham (JC) virus, but also to herpetic and varicella zoster encephalitis^18^. Hence, varicella zoster vaccine should be considered for other immunosuppressants and not only S1P receptor modulators. 

Hepatitis B virus reactivation is five times higher in patients under rituximab when compared with other immunosuppressive therapies^19^. Indeed, in patients with multiple sclerosis, ocrelizumab was associated with reactivation of hepatitis B. Of note, CD-20 cell depletion impairs the B-cell dependent CD8 T-cell activation, reducing the cytotoxic defense against the hepatitis B virus^20^. Also, an immunological environment with suboptimal humoral and cellular responses to hepatitis B virus promotes viral replication and can cause immune-escape mutations^21^.

Hepatitis B vaccines derive from synthetic antigens developed from nucleotide sequence data to prepare large quantities of proteins by recombinant DNA technology^11^. Synthetic antigen vaccines are considered to be safe in patients using immunosuppressants. Current guidelines recommend that patients should test an Anti-HBs antibody prior to the initiation of anti-CD20 drugs. Patients with Anti-HBs negative antibodies should receive an additional jab of hepatitis B vaccine. However, patients with Anti-HBs antibody titers superior to 10 UI/mg should not receive an additional vaccine, but if a positive anti-HBc antibodies is found, antiviral prophylactic treatment should be considered to prevent hepatitis B virus reactivation for at least 12 months after the discontinuation of the immunotherapy^22^.

Case studies have suggested an association of the use of Fingolimod with HPV infection such as cutaneous warts, cervical and anogenital cancers^23^. The reduced immune response to viral infections and potential impaired cancer cells surveillance in patients receiving Fingolimod may explain this association^23^. Case studies have also described rapid progression of low-grade cervical dysplasia into invasive cancer in patients while on natalizumab therapy^24^, suggesting that cell adhesion inhibition might also compromise mucocutaneous defenses.

 HPV vaccines are synthetic antigen vaccines produced by recombinant DNA technology and are considered safe in patients with CNSDD. Current guidelines recommend that patients aged between nine and 26, of either gender, should receive three doses (0, 2 and 6 months) of the HPV vaccine. Annual screening for cervical cancer should be considered for patients receiving immunosuppressants^23^.

The normal immune response to respiratory viruses such as SARS-CoV-2 and influenza are compromised in DMDs^25^. The production of effective neutralizing antibodies is not only affected by B-cell depleting agents^26^, but also by other immunotherapy strategies that compromise T-cell mediated B-cell activation such as Fingolimod^27^. The latter may also compromise the cytotoxic activation necessary for virus neutralization and eradication.

Current guidelines recommend that patients with CNSDD should receive an annual dose of the influenza vaccine. Vaccine against influenza is an inactivated vaccine and is considered safe in patients with CNSDD. However, current influenza vaccines do not induce broadly neutralizing antibodies that recognize multiple strains of the virus and thus short-life antibody-mediated protection explains why the vaccines should be administered annually^28^ ^,^ ^29^. 

The management of patients with CNSDD during the COVID-19 pandemic was challenging. Multicentric observational retrospective studies demonstrated that patients with CNSDD presented a higher likelihood of hospitalization and intensive care unit admission compared with the general Brazilian population^30^. Different strategies such as telemedicine^31^ and oral methylprednisolone pulse therapy for relapses^32^ were performed, but no strategy was as successful as immunization. 

Vaccines against SARS-Cov-2 can be inactivated (e.g. Coronavac), live viral vaccines involving recombinant non-replicant viruses (e.g. Astrazeneca), and RNA vaccines (e.g. Pfizer, Jansen). Live viral vaccines involving recombinant non-replicant virus have the advantage of inducing a full immune response similar to what occurs with live attenuated virus vaccines, but address safety concerns of live attenuated virus vaccines as they use a non-replicating vector. RNA vaccines activate RNA sensors and are amplified by lipid nanoparticles that act as adjuvant to enhance the immune response. In conclusion, all these technologies appear to be safe in patients with CNSDD. Multiple doses of these vaccines are recommended to allow a long-term response and the best schedule is still unknown.

**Figure 2.**
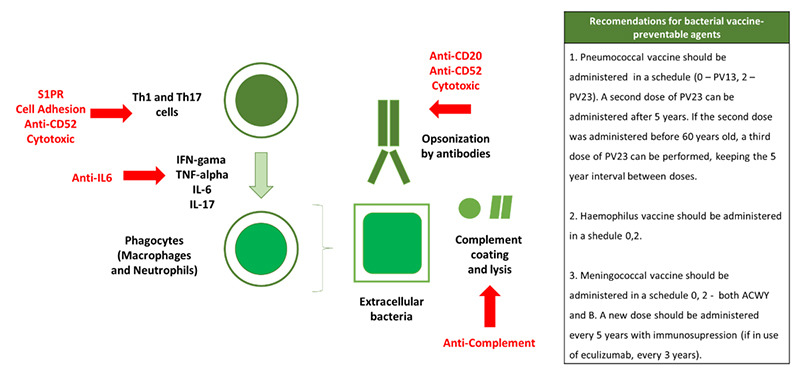
Normal immune response to bacterial agents, how disease modifying drugs impact this response and what vaccine strategies we should implement.

The three most relevant bacterial vaccine-preventable agents in patients with CNS demyelinating diseases are *Pneumococcus*, *Haemophilus,* and *Meningococcus*. Figure 2 summarizes the normal immune response to bacterial agents, the impact of DMDs and the vaccine recommendations in clinical practice.

The normal immune response to extracellular bacteria involves phagocytosis. Phagocytosis is amplified by other elements of the innate immune system, such as the C3b coating of bacteria, that allows for pore formation and consequently the lysis of microbes, a process mediated by the complement pathway. Phagocytosis is also amplified by the humoral response due to the opsonization and Fc receptor-mediated phagocytosis. Furthermore, adaptive cellular response, mediated by T CD4 lymphocytes, improves phagocytosis due to the intense release of pro-inflammatory cytokines such as IFN-gamma, IL-17, TNF-alpha^11^.

 Patients with multiple sclerosis are found to be at an increased risk of the use of antibiotics in population studies^1^, particularly with anti-CD20 monoclonal antibodies such as rituximab.

 Anti-CD20 monoclonal antibodies can increase the risk of mild and severe respiratory infections because of secondary hypogammaglobulinemia and the reduction of the circulating B-cell dependent T-cell activation associated with cytokine release and antigen presentation^33^. Two patients died from pneumonia in the randomized clinical trial that evaluated ocrelizumab in primary progressive multiple sclerosis^34^. Large observational studies demonstrated that additional pneumococcal and haemophilus B vaccines can reduce the risk of respiratory infections in patients under rituximab^35^.

 Pneumococcal vaccine (PV) can be composed of polysaccharide antigens or conjugated vaccine (polysaccharide coupled with protein). As polysaccharides are T-independent antigens, they tend to elicit low-affinity antibody responses and are best suited for boosters although not the first dosage. For this reason, it is recommended that the first dosage of pneumococcal vaccine should be PV13 vaccine (conjugated vaccine) with a booster dose of PV23 vaccine (polysaccharide vaccine), two months later^36^.

Eculizumab is an anti-C5 monoclonal antibody considered as a treatment option for neuromyelitis optica, preventing the aquaporin-4 mediated complement-induced damage^37^. This drug causes a defective bactericidal complement activity used against encapsulated bacteria. The impact of deficient C5a-C9 is more evident in invasive meningococcal infections, leading to a 10,000-fold increase in the risk of invasive infection and an estimated meningococcal disease incidence of 1.5%^38^. To mitigate this risk, it is recommended to perform additional boosters of meningococcal vaccine MenACWY every five years and of MenB every two to three years as long as an increased risk of meningococcal infection persists^39^ ^,^ ^40^. 

### Which vaccines should not be administered in patients with CNSDD?

 CNSDD is considered a disease of autoimmune phenomenon that occurs in genetically susceptible individuals who face environmental factors. As vaccines can induce autoimmune neurological adverse events, previous studies evaluated whether vaccines could induce an overall excess of cases of CNSDD.

In a large case-control study, vaccination against hepatitis B, influenza, tetanus, measles, or rubella was not associated with increased risk of multiple sclerosis or optic neuritis^41^. Systematic reviews confirmed that vaccines were not associated with central nervous system demyelination^42^ ^,^ ^43^. Hence, no vaccine should be avoided as a public health concern of developing a CNSDD. 

 Another concern is that vaccines can induce relapse in patients who already present CNSDD. Systematic reviews from the American Academy of Neurology^29^ and the French Society of Neurology^28^ found no association between vaccines and relapses in patients with multiple sclerosis. Even for the recent COVID-19 vaccines, cohort studies did not demonstrate an increased risk of relapse activity^44^.

 One exception to this rule is the yellow fever vaccine, a weak form of the virus, which was associated with increased relapse rate in travelers with multiple sclerosis^29^. However, these studies were limited by small-size samples and further research is needed.

 In patients with neuromyelitis optica, vaccines were associated with relapse in observational retrospective studies^45^. Particularly, in this study, the risk was found to be low overall, estimated at 3% of patients, and persisting for 90 days following vaccination. However, this risk was not increased when patients were on adequate immunotherapy. Also the average risk of relapse was lower in adequately vaccinated patients than in patients who did not receive vaccination, possibly because infections can also trigger relapses.

 The safety of live vaccinations in patients receiving immunotherapy is another concern. A systematic review^46^ including different immunosuppressed patients demonstrated that although the administration of live vaccines was safe in most patients, some can develop serious vaccine-related adverse events such as infection with the vaccine strain. The live vaccines that should be avoided in patients with CNSDD receiving DMDs include: mumps, measles, rubella, yellow fever, varicella vaccine/herpes zoster, oral polio, rotavirus, oral typhoid, smallpox and Bacillus Calmette-Guérin (BCG).

### When is the best time to administer vaccines in patients with CNSDD?

**Figure 3.**
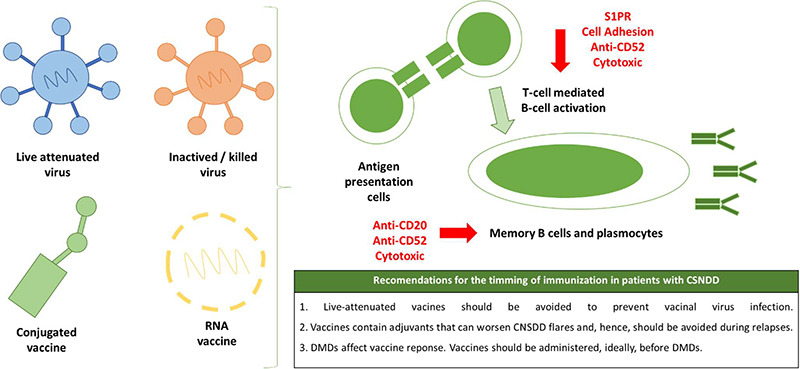
Normal vaccine response and vaccine recommendations related to contraindications and timing.

 Most vaccines work by inducing neutralizing antibodies. Vaccine-induced neutralizing antibodies are produced by long-life plasma cells and memory B-cells. These aspects of humoral response are best induced in the germinal center reaction, which requires cytokines and antigen presentation provided by protein antigen-specific CD4+ T follicular helper cells^11^. Figure 3 summarizes the normal vaccine-induced response, how DMDs impact this process and recommendations for clinical practice to optimize this process.

Anti-CD20 monoclonal antibodies used in CNSDD can induce transitory or permanent depletion of multiple B-cell lineages and impair the production of neutralizing antibodies. In the VELOCE study^26^, the humoral response to non-live vaccines in patients with multiple sclerosis receiving ocrelizumab was attenuated when compared with patients receiving interferon beta or no immunotherapy.

As the vaccine-induced humoral response is optimized by the cellular response, it is expected that other DMDs may also impact the vaccine effectiveness. The S1P receptor modulation provided by Fingolimod compromises the cellular response against the vaccine antigens and, hence, impairs the T-cell-mediated activation of B-cells. In a populational study in England, the risk of SARS-Co-2 infection in patients with multiple sclerosis on Ocrelizumab or Fingolimod were increased when compared to the general population despite mass vaccination^47^.

 Therefore, the best time to administer vaccines in patients with CNSDD is before the introduction of any immunomodulatory therapy or at the end of the last dose of intermittent immunotherapies. For instance, for rituximab, the optimal timing for vaccination is at least three months after the last dose and at least one month before the next dose.

 However, humoral responses after vaccines peaks after two to four weeks. For this reason, most guidelines recommend that vaccines should be administered up to four weeks before the start of the next dose of DMDs. Vaccines are usually contraindicated during flare-ups of CNSDD, despite the lack of studies on this subject. In an observational retrospective study, vaccines were associated with relapses in patients with neuromyelitis optica who are not on preventive immunotherapy but not among patients receiving adequate immunotherapy^45^. A possible explanation is that adjunctives used in vaccines may increase the activity of autoreactive B and T lymphocytes causing flare-ups of CNSDD, particularly when immunotherapy is suboptimal such as during relapses. We provide a comparison of current guidelines regarding vaccination in patients with CNSDD in Table 1. 

**Table 1.** Comparison of current guidelines regarding vaccination in patients with CNSDD.

	French Multiple Sclerosis Society	American Academy of Neurology (AAN)	Brazilian Committee for Treatment and Research in Multiple Sclerosis (BCTRIMS)
Which vaccines should be administered in patients with CNSDD?	Vaccination schedule associated with annual influenza vaccine	Clinicians should recommend that patients with MS follow all local vaccine standards, unless there are specific contraindications. Clinicians should recommend that patients with MS receive the influenza vaccination annually.	Check the vaccines according to the existing vaccines available in the Brazilian National Immunization Program (NIP)
Which vaccines should not be administered in patients with CNSDD?	Vaccines do not cause MS or MS relapse Live-attenuated vaccines are contraindicated	Clinicians should recommend against using live-attenuated vaccines in people with MS receiving immunosuppressive therapies.	It is also important to note that vaccines are safe, and physicians should encourage their use in all patients. Special care should be taken when live attenuated viruses are involved.
When is the best time to administer vaccines in patients with CNSDD?	The vaccination status should be verified before DMDs are introduced	Vaccinate patients four to six weeks before DMDs Clinicians should delay vaccinating people with MS who are experiencing a relapse.	The DCNI/ABN and BCTRIMS recommend that patients with CNS demyelinating diseases (e.g., MS and NMOSD) be continually monitored for updates to their vaccination schedule, especially at the beginning or before a change in treatment with a disease modifying drug (DMD)

In conclusion, infections are the main cause of death in patients with CNSDD and can be prevented by vaccines. The evaluation of vaccine-preventable viruses should include varicella zoster, hepatitis B, HPV, influenza, and Sars-Cov-2. The evaluation of vaccine-preventable bacteria should include pneumococcus, haemophilus and meningococcal. Live-attenuated (e.g. measles or yellow fever) vaccines should be avoided. Vaccines should not be administered during flare-ups or, if possible, before the initiation of disease-modifying agents.
